# Importance of Genetic Testing in Children With Generalized Epilepsy

**DOI:** 10.7759/cureus.59991

**Published:** 2024-05-09

**Authors:** Madison Heebner, Gayatra Mainali, Sharon Wei, Ashutosh Kumar, Sunil Naik, Sandeep Pradhan, Prakash Kandel, Jaclyn Tencer, Paul Carney, Sita Paudel

**Affiliations:** 1 Neurology, Penn State College of Medicine, Hershey, USA; 2 Pediatric Neurology, Penn State Health Milton S. Hershey Medical Center, Hershey, USA; 3 Neurology, Penn State University, Hershey, USA; 4 Biostatistics, Penn State College of Medicine, Hershey, USA; 5 Pediatrics and Neurology, University of Missouri, Columbia, USA

**Keywords:** epilepsy panel, childhood absence epilepsy, genetic panel testing, diagnostic genetic testing, pediatric neuroimaging, generalized epilepsy, childhood epilepsy

## Abstract

Introduction: Epilepsy is a neurological disorder characterized by the predisposition for recurrent unprovoked seizures. It can broadly be classified as focal, generalized, unclassified, and unknown in its onset. Focal epilepsy originates in and involves networks localized to one region of the brain. Generalized epilepsy engages broader, more diffuse networks. The etiology of epilepsy can be structural, genetic, infectious, metabolic, immune, or unknown. Many generalized epilepsies have presumed genetic etiologies. The aim of this study is to compare the role of genetic testing to brain MRI as diagnostic tools for identifying the underlying causes of idiopathic (genetic) generalized epilepsy (IGE).

Methods: We evaluated the diagnostic yield of these two categories in children diagnosed with IGE. Data collection was completed using ICD10 codes filtered by TriNetX to select 982 individual electronic medical records (EMRs) of children in the Penn State Children’s Hospital who received a diagnosis of IGE. The diagnosis was confirmed after reviewing the clinical history and electroencephalogram (EEG) data for each patient.

Results: From this dataset, neuroimaging and genetic testing results were gathered. A retrospective chart review was done on 982 children with epilepsy, of which 143 (14.5%) met the criteria for IGE. Only 18 patients underwent genetic testing. Abnormalities that could be a potential cause for epilepsy were seen in 72.2% (13/18) of patients with IGE and abnormal genetic testing, compared to 30% (37/123) for patients who had a brain MRI with genetic testing.

Conclusion: This study suggests that genetic testing may be more useful than neuroimaging for identifying an etiological diagnosis of pediatric patients with IGE.

## Introduction

Epilepsy is a neurological disorder characterized by the predisposition for recurrent unprovoked seizures. It can broadly be classified as focal, generalized, combined focal and generalized, or unknown depending on the patient’s seizure types [1]. Patients with generalized epilepsy have generalized seizures that have simultaneous onset in networks involving the bilateral hemispheres. The etiology of epilepsy can be structural, genetic, infectious, metabolic, immune, and unknown. 

While the classification of epilepsy syndromes has been a dynamic process over several years, in 2017, the International League Against Epilepsy (ILAE) described the genetic generalized epilepsies (GGEs), which are characterized by generalized seizure types that show generalized interictal spike-and-wave discharges on EEG with a presumed genetic etiology. Included in this broader category are the idiopathic (genetic) generalized epilepsies (IGEs), which are further delineated into childhood absence epilepsy (CAE), juvenile absence epilepsy (JAE), juvenile myoclonic epilepsy (JME), and epilepsy with generalized tonic-clonic seizures alone (GTCA) [1]. These epilepsies are the most common syndromes within the GGEs, often have a favorable prognosis in regard to seizure control and developmental outcome, and frequently have overlapping features among each other [2].

While epilepsies can have very variable etiologies, the IGEs are thought to have a genetic basis with a complex inheritance pattern [3]. Given this complexity, the yield of pathogenic gene identification in IGEs has been relatively low; however, monogenic pathogenic variants (e.g., SLC2A1, GABRA1, and GABRG2) and copy number variants (e.g., 15q13.3 microdeletions) have been identified in some patients [4-9]. There have also been studies specifically suggesting that ultra-rare genetic variation in GABAergic inhibition pathways underlie various forms of epilepsy including GGEs [10]; however, other studies evaluating single rare variants did not find statistically significant variants in patients with IGE versus those in controls [11].

Diagnosis of IGE mostly consists of history taking, physical and neurological exam, EEG, and sometimes brain imaging. These steps are taken to help rule out common differentials, such as syncope and psychogenic non-epileptic seizures, and to make an accurate diagnosis [12,13]. However, even with this current structure, some seizures are difficult to diagnose, such as absence seizures [14].

While brain imaging in some patients with generalized epilepsy may show a cerebral abnormality [6], in most cases of generalized epilepsy, there does not tend to be an associated cerebral structural finding on brain imaging. Per the ILAE, neuroimaging in the IGEs is expected to be normal and not typically indicated unless there are atypical features, drug-resistant seizures, or persistent slowing on EEG [2]. Gaillard et al. in 2009 had similarly published guidelines suggesting that neuroimaging is not universally required in typical IGE [15]. Betting et al. evaluated 134 patients with IGE and 24% showed abnormalities, 88% of which were nonspecific. In the patients who did have more potentially significant findings, the findings were not always considered causative and often those patients had a higher proportion of EEG focality, which is often concordant with the location of MRI abnormalities [13,15-16]. Despite the relatively low yield of brain imaging in this patient population, the frequency of brain imaging as a part of the diagnostic workup in these patients has remained significant [6]. In the pediatric population, neuroimaging has the added consideration of the need for sedation for imaging typically for patients younger than seven years old.

There is significant practice variation regarding the evaluation of patients with IGEs and a lack of clear consensus among providers regarding genetic evaluation and neuroimaging. Genetic testing may be a more efficient way to help in the diagnostic process for patients with IGE. This may allow for a faster diagnosis and therefore an earlier start on treatment planning, which requires an integrated approach by interprofessional healthcare members and coordination with the patient and family.

Neuroimaging can help to rule out obvious structural causes and determine surgical planning if an operable lesion is identified, but the relatively high rate of negative results leaves many patients without an identifiable cause for their seizures [17]. If genetic testing has a higher diagnostic yield when compared to neuroimaging, it may be a useful tool to assist in determining more precise etiologies of IGE.

The aim of this study was first to analyze our group's practice regarding diagnostic testing for patients with IGE, specifically in terms of genetic and neuroimaging testing, and, second, to analyze the yield of these modalities for our patients in identifying an underlying etiology for the epilepsy.

This article was previously presented as a meeting abstract at the 46th Annual Meeting of the Southern Pediatric Neurology Society on March 11, 2023.

## Materials and methods

This was a retrospective chart review study done after Institutional Review Board (IRB) approval from the Penn State University, Human Research Protection Program (HRPP) (FWA00004251, IRB #: STUDY00017431). Data collection was completed by filtering charts by ICD10 codes and compiling them with TriNetX, a research population cohort search tool. All providers in the Penn State Children’s Hospital at the time of the TriNetX query were included. This search yielded 982 individual electronic medical records (EMRs) of children (<18 years) with a history of IGE at Penn State Children’s Hospital. Medical records from 2011 to 2020 were pulled for this study. To confirm the diagnosis of IGE, diagnostic data including clinical history and EEG reports were reviewed. Data from these patients with IGE were collected to determine if these patients underwent neuroimaging and/or genetic testing. Genetic testing, such as chromosomal microarrays, the (Behind the Seizure) epilepsy gene panel, and whole-exome sequencing (WES), were included.

Inclusion criteria were children under 18 years of age with a diagnosis of IGE who had EEG findings of generalized spike and wave complexes of 4-6 hz or 3 hz spike-wave complexes with an otherwise normal background and who underwent neuroimaging, genetic testing, or both [17]. Patients with other types of epilepsy, such as focal or unclassified epilepsy; patients with EEG findings, such as slow spike and waves suggestive of a Lennox-Gastaut pattern or with a significantly abnormal background; or patients with other significant neurological comorbidities that are associated with MRI abnormalities, such as cerebral palsy, autism, or intellectual disabilities, were excluded from the study [18-19]. Patients had to meet all inclusion criteria to be included in the study, and patients meeting any exclusion criteria were not included.

To analyze the data, we compared demographic and clinical characteristics using χ2, t-tests, or Mann-Whitney tests, depending on the data type and normality. Demographic and clinical characteristics were summarized using means and standard deviations (SDs) for continuous variables and counts and percentages for categorical variables. The receiver operating characteristic (ROC) curve analysis was used to test the diagnostic accuracy of genetic testing and MRI. To compare the diagnostic capability of different imaging modalities, the area under the ROC curve (AUROC) was used to assess the ability to predict disease accurately [20-22]. The AUC is an effective way to summarize the overall diagnostic accuracy of the test. It takes values from 0 to 1, where a value of 0 indicates a perfectly inaccurate test, and a value of 1 reflects a perfectly accurate test. The AUC can be computed using the trapezoidal rule [22]. In general, an AUC of 0.5 suggests no discrimination (i.e., ability to diagnose patients with and without the disease or condition based on the test), 0.7 to 0.8 is considered acceptable, 0.8 to 0.9 is considered excellent, and more than 0.9 is considered outstanding [21]. A p-value <0.05 was considered significant. Statistical analyses were performed using the SAS statistical package (version 9.4, SAS Institute, Inc., Cary, NC, USA).

## Results

**Figure 1:**
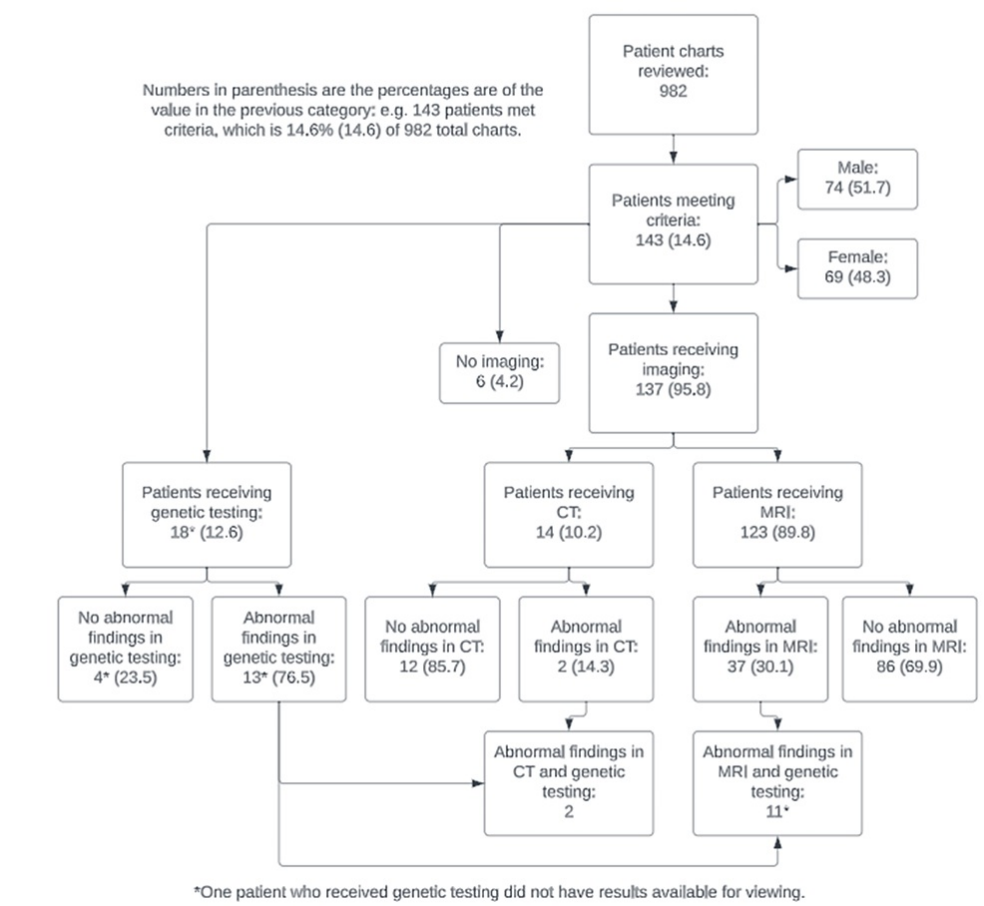
Patient demographic flowchart CT: computed tomography scan, MRI: magnetic resonance imaging scan

Of the 982 children with epilepsy, 143 (14.5%) met the criteria for IGE and were included in this study (Figure 1). The median age of the participants was eight years (interquartile range (IQR): four to 12 years), and 69 (48%) were female. Subject demographics are summarized in Table 1 and Table 2. Among them, 95.8% (137/143) had neuroimaging (head CT or MRI brain), and six (4.2%) did not have any form of neuroimaging. A total of 123 patients underwent an MRI. The MRI findings reveal that 14 patients had hyperintense lesions/signal abnormalities, two had developmental anomalies/cysts, and 21 had other non-diagnostic findings, such as slight asymmetry in the hippocampus or prominent perivascular spaces. In other words, 30% (37/123) of the patients had abnormal findings but were overall nonspecific or did not help to identify the etiology of seizures.

**Table 1:** Patient demographics IQR: interquartile range, CT: computed tomography scan, MRI: magnetic resonance imaging scan

Patient Demographics	Total (N = 143)
Age at Diagnosis	
N	133
Mean (SD)	8.2 (4.25)
Median	8.0
IQR	4.0, 12.0
Sex, n (%)	
Female	69 (48.3%)
Male	74 (51.7%)
Types of imaging, n (%)	
No imaging	6 (4.2%)
MRI	123 (86.0%)
CT	14 (9.8%)
Abnormal finding in the scan, n (%)	
No	98 (71.5%)
Yes	39 (28.5%)
Abnormal finding in CT, n (%)	
No	12 (85.7%)
Yes	2 (14.3%)
Abnormal finding in MRI, n (%)	
No	86 (69.9%)
Yes	37 (30.1%)
Genetic testing, n (%)	
No	125 (87.4%)
Yes	18 (12.6%)
Abnormal finding in genetic testing, n (%)*	
No	4 (22.2%)
Yes	13 (72.2%)
Types of genetic testing in patients with abnormal findings, n (%)*	
Chromosomal microarray	2 (15.4%)
Epilepsy gene panel	8 (61.5%)
Whole exome sequencing	2 (15.4%)
Not documented	1 (7.7%)
Types of epilepsy, n (%)	
Childhood absence epilepsy (CAE)	27 (19.1%)
Generalized tonic-clonic seizures alone (GTCA)	25 (17.7%)
Juvenile absence epilepsy (JAE)	13 (9.2%)
Juvenile myoclonic epilepsy (JME)	15 (10.6%)
Generalized epilepsy	61 (43.3%)
Abnormal MRI brain findings, n (%)	
Hyperintense lesion/signal abnormalities	14 (37.8%)
Developmental anomalies/cyst	2 (5.4%)
Others	21 (56.8%)
Modality, n (%)	
MRI only	107 (74.8%)
CT only	12 (8.4%)
MRI + genetic testing	16 (11.2%)
CT + genetic testing	2 (1.4%)
No testing	6 (4.2%)
*Of the 18 patients who had genetic testing, one had results that were not available in the EMR. Therefore, it is unknown whether this patient’s genetic testing was normal or abnormal.	

**Table 2:** Patient demographics by testing type (genetic testing vs. MRI) IQR: interquartile range, CT: computed tomography scan, MRI: magnetic resonance imaging scan

Patient demographics by testing type (genetic testing vs. MRI)	Types of testing			
	CT only (N = 12)	Genetic* (N = 18)	MRI only (N = 107)	P-value
Age at diagnosis				0.0004^1^
N	12	13	107	
Mean (SD)	11.6 (3.63)	4.8 (3.60)	8.2 (4.16)	
Median	12.5	4.0	8.0	
IQR	10.0, 14.0	3.0, 7.0	5.0, 12.0	
Sex, n (%)				0.8257^2^
Female	5 (41.7%)	8 (44.4%)	53 (49.5%)	
Male	7 (58.3%)	10 (55.6%)	54 (50.5%)	
Abnormal finding in the scan, n (%)				0.4816^2^
No	10 (83.3%)	14 (77.8%)	74 (69.2%)	
Yes	2 (16.7%)	4 (22.2%)	33 (30.8%)	
Abnormal finding in the MRI, n (%)				
No	0 (.%)	12 (75.0%)	74 (69.2%)	
Yes	0 (.%)	4 (25.0%)	33 (30.8%)	
Genetic testing, n (%)				<.0001^2^
No	12 (100.0%)	0 (0.0%)	107 (100.0%)	
Yes	0 (0.0%)	18 (100.0%)	0 (0.0%)	
Abnormal finding in the genetic testing, n (%)				
No	0 (.%)	4 (22.2%)	(100.0%)	
Yes	0 (.%)	13 (72.2%)	0 (0.0%)	
Types of epilepsy, n (%)				0.0337^2^
Childhood absence epilepsy (CAE)	1 (9.1%)	3 (17.6%)	23 (21.5%)	
Generalized tonic-clonic seizures alone (GTCA)	1 (9.1%)	3 (17.6%)	19 (17.8%)	
Juvenile absence epilepsy (JAE)	0 (0.0%)	1 (5.9%)	11 (10.3%)	
Juvenile myoclonic epilepsy (JME)	1 (9.1%)	6 (35.3%)	8 (7.5%)	
Generalized epilepsy	8 (72.7%)	4 (23.5%)	46 (43.0%)	
Abnormal MRI brain findings, n (%)				
Hyperintense lesion/signal abnormalities	0 (.%)	1 (25.0%)	13 (39.4%)	
Developmental anomalies/cyst	0 (.%)	0 (0.0%)	2 (6.1%)	
Others	0 (.%)	3 (75.0%)	18 (54.5%)	
^1^Wilcoxon-Rank p-value; ^2^Fisher's exact p-value *All patients who received genetic testing also received neuroimaging.				

Genetic testing was done in 18 (12.6%) patients; 13 (72.2%) of these patients had genetic abnormalities discussed in Table 3. Table 3 only shows 12 abnormalities since there was no specification on what abnormalities were shown for one of the patients.

**Table 3:** Genetic abnormalities

Specific type of gene abnormality	Variant	Genetic testing results
SCN1A	Deletion (Exon 1)	Pathogenic
SCN9A gene	c.1643G>1 (p.Arg548Gln)	Variant of unknown significance
NSUN2	2 variants: One variant p.W377X autosomal recessive and second variant p.Q53X, autosomal recessive	Likely pathogenic variant
Duplication 17q12	1.82Mb Gain from 17q12	Pathogenic
Y - POLG NM_001126131.1c.752C>T	001126131.1c.752C>T	Heterozygous pathogenic
TASR2	c.773C>T (p.Ser258Leu)	Pathogenic
ALG1	c.773C>T	Pathogenic
KCNA1	c. 450G>T	Variant of unknown significance
7q36.1	Deletion	Variant of unknown significance
NONO gene	Not available	Pathogenic
IQSEC2	Not available	Pathogenic
SLC2A1 gene	c.875delA	Pathogenic

In the first part of the analysis, the genetic testing and MRI group were compared using the Wilcoxon rank-sum test or Fisher's exact test. Statistical significance was not reached between the MRI and genetic testing groups for any variables. Although non-significant, the MRI group had a higher proportion of hyperintense lesion/signal abnormalities (1 (25.0%) vs. 13 (39.4%)) and developmental anomalies/cysts (0 (0.0%) vs. 2 (6.1%)). None of them were statistically significant at p < 0.05.

**Figure 2:**
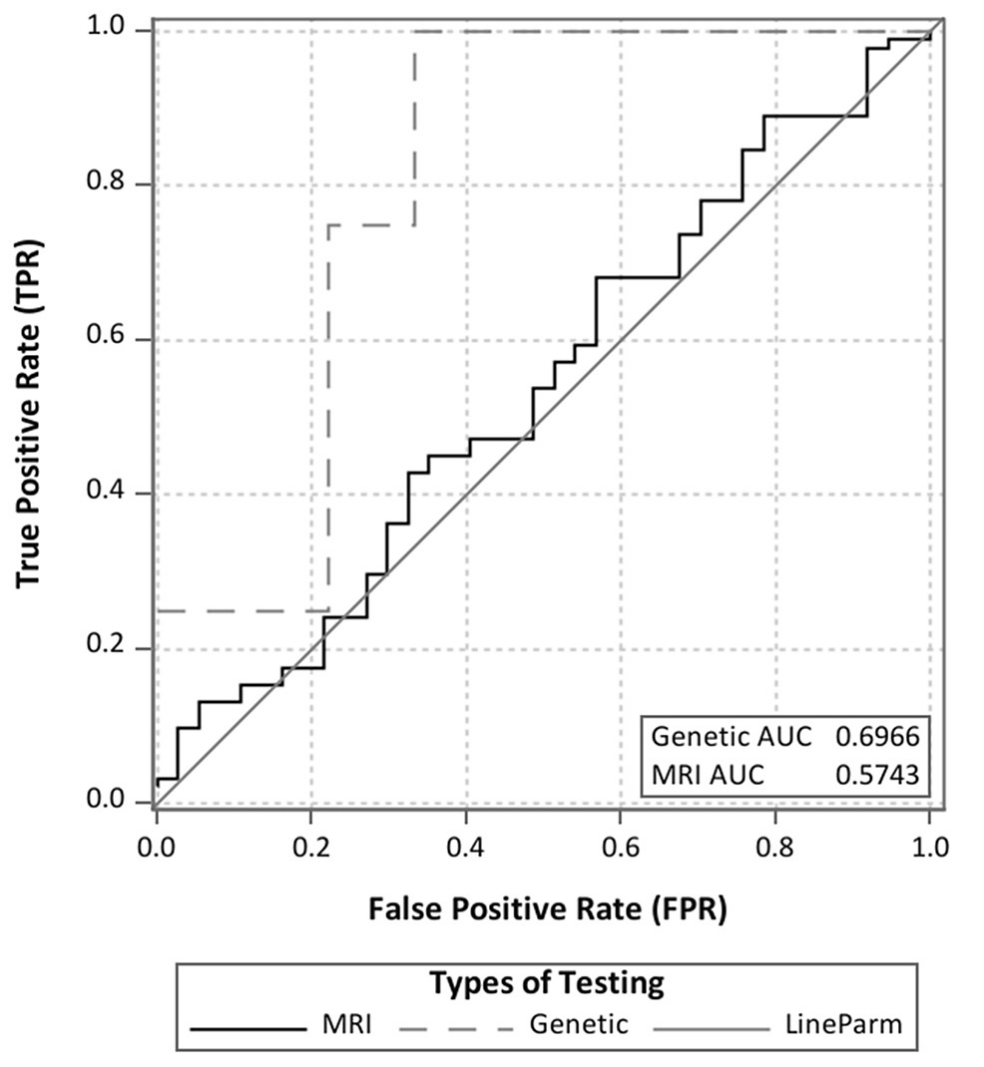
Area under ROC curve comparison for MRI and genetic testing

As sensitivity and specificity values may vary when different cut-off points are taken, an ROC curve analysis was performed to quantify the diagnostic accuracy of MRI or genetic testing, either individually or combined with a scan [22]. The ROC curves for MRI and genetic testing were derived individually to identify potential etiologies for IGE (Figure 2), and the corresponding AUCs with 95% confidence intervals (CIs) were calculated. Both tests showed less discriminatory power for identifying potential etiologies for IGE (AUC genetic testing: 0.6955 (95% CI: 0.01 >999.9)), AUC MRI: 0.574 (95% CI: 0.707 15.616)). Genetic testing has a fairly acceptable capability to identify potential etiologies for generalized epilepsy compared to MRI, which suggests slight discrimination power.

## Discussion

According to previous studies, genetic studies like WES, next-generation sequencing (NGS), and chromosomal microarray (CMA) are found to be efficient for the etiological diagnosis of epilepsy especially for early-onset epileptic encephalopathy. The diagnostic yield of multigene epilepsy panels is around 18% to 28% [23-24].

The yield of genetic testing is unclear from previous studies in patients with IGE with normal development. However, according to the study by Mercimek-Mahmutoglu et al. in patients with global developmental delay and intractable epilepsy, 28% of the 110 patients had genetic abnormality, of which 7% had inherited metabolic disorders, 21% had other genetic causes including genetic syndromes, pathogenic copy number variants, and mutations in the SCN1a, SCN2A, SCN8A, KCNQ2, STXBP1, PCDH19, and SLC9A6 genes contributing to their epileptic encephalopathy [25]. In our study, we wanted to compare genetic test results with commonly done brain MRIs in children with IGE to find out which test is better at detecting potential sources for epileptic seizures or if these tests complement each other.

In our study, we found that 18 children (<18 years) with IGE had genetic testing, and abnormalities were found in 13 of the genetic testing studies (72%, 13/18). A brain MRI was done in 123 children, and some abnormality was found in 37 of the imaging studies (30%, 37/123). We identified that most MRI findings were white matter hyperintensities, small cysts, an unspecific asymmetry of the hippocampus, prominent vascular markings, or other non-specific findings. Regarding genetic testing, each IGE patient had separate abnormalities. Some abnormalities were pathogenic gene mutations, and others were variants of unknown significance.

The MRI abnormalities seen were non-diagnostic regarding the cause of epilepsy and did not appear to relate to the patient’s symptoms with respect to presentation or EEG results. In other words, the patient’s seizures were not explained by the results of the MRI. While few of the genetic testing results revealed variants of unknown significance, the rate of genetic abnormalities in patients with IGE was higher than the rate of abnormalities in neuroimaging, indicating that genetic abnormalities may be more common than structural abnormalities visualizable on neuroimaging within this patient population.

There were some limitations to our study. Our sample size was limited. Slightly under 1000 charts were reviewed, which yielded under 150 eligible participants. Our patient population was also limited, as the participants were selected from chart data within the Penn State Hershey Children’s Hospital system only. Of the eligible patient population, genetic testing was performed in only 18 patients whereas neuroimaging was performed in 137 patients, which could also potentially confound the result. One reason for the small patient population may have been because the Behind the Seizure panel, which was used for epilepsy gene testing at our institution, was only made free for patients under the age of eight beginning in 2019-2020 [26].

## Conclusions

Increasing the diagnostic yield of epilepsy studies may be important to assist with future management and more individualized treatment of the disease. Furthermore, most of the patients in this study received neuroimaging than genetic testing. Neuroimaging is commonly used after a seizure to detect an underlying cause, such as a structural abnormality or lesion, and can guide surgical management if so indicated; however, many neuroimaging studies are negative for obvious seizure-causing processes mainly for patients with IGE. Many of these patients also required anesthesia for neuroimaging, which potentially can have its own complications. 

This study suggests genetic testing is probably more helpful for etiological diagnosis compared to brain MRI in patients with IGE. Given the small sample size and the potential for selection bias, a larger study would likely be helpful to clarify further.
